# Urethral multiplicity in boys: systematic review of case reports and case series from the last 15 years

**DOI:** 10.3389/fped.2024.1404947

**Published:** 2024-06-11

**Authors:** Horea Gozar, Zsolt Bara, Evelyn Kovacs, Iulia Gozar, Zoltan Derzsi

**Affiliations:** ^1^ Clinic of Pediatric Surgery and Orthopedics, County Emergency Clinical Hospital, Târgu Mureș, Romania; ^2^ Department of Pediatric Surgery and Orthopedics, George Emil Palade University of Medicine, Pharmacy, Science and Technology of Târgu Mureș, Târgu Mureș, Romania; ^3^ University of Agricultural Sciences and Veterinary Medicine of Cluj-Napoca, Cluj-Napoca, Romania

**Keywords:** urethral duplications, urethral multiplicity, urethral triplication, prepubic sinus, voiding

## Abstract

**Introduction:**

Urethral multiplicity is a rare congenital anomaly characterized by the presence of two or more urethral channels. It is more common in males and can cause double urinary stream, incontinence, obstruction, and recurrent urinary infections. Diagnosis is difficult due to diverse clinical manifestations. Implementing an evidence-based treatment plan is challenging due to the need for more concise and informative summary publications. Our paper provides a comprehensive review of the management of this pathology and might serve as a valuable resource for pediatric urologists and specialists in the field.

**Methods:**

A comprehensive search in four electronic databases, PubMed®, PubMed Central® (PMC), Scopus, and Clarivate Analytics's Web of Science (WoS), was conducted to identify case reports and series published between 2008 and 2023 on urethral multiplicities. The quality of the articles was assessed using qualified instruments. Covidence® tool-guided synthesis was followed by individual patient data extraction. Further classifications and analysis were made using Microsoft Excel®.

**Results:**

Out of the 90 papers included in the review, 62 were case presentations, and 28 were case series. We found 250 boys with urethral multiplicity. Based on Effman's classification, there were 38 cases of type I (15.3%), 21 type IIA1 (8.4%), 55 type IIA2 (22.1%), 91 type IIA2Y (36.5%), 4 type IIB (1.6%), and 6 type III (2.4%) urethral duplications. There were 19 cases of prepubic sinuses (7.6%), 9 triplications (3.6%), and 6 unknown forms (2.4%). We have provided data for each type, including clinical presentation, investigations, surgical management, and outcomes.

**Conclusions:**

Urethral multiplicities are a rare and varied group of malformations that require high-quality imaging examination for successful management. Treatment is specific to each patient and may depend on the surgeon's preference or skill.

**Systematic Review Registration:**

https://www.crd.york.ac.uk/prospero/display_record.php?ID = CRD42023471685, identifier (CRD42023471685).

## 1 Introduction

Specialists treating patients with urethral multiplicity may face unique challenges. Given the rarity of this congenital anomaly, diagnosis and classification can be difficult due to its diverse range of manifestations. The available articles and case reports often contain ambiguous and conflicting information, thereby accurate diagnoses and effective treatment based only on the available literature are difficult.

Urethral multiplicity is a congenital anomaly characterized by two or more urethral channels with variable location and extension. Besides urethral duplications, other types of multiplicities include the presence of prepubic sinuses and urethral triplications. They are more common in males and usually occur in the sagittal plane. Patients may be asymptomatic or symptomatic, with typical clinical findings including incontinence, obstruction, recurrent urinary infections, and occasionally a double urinary stream (1).

**Figure 1:**
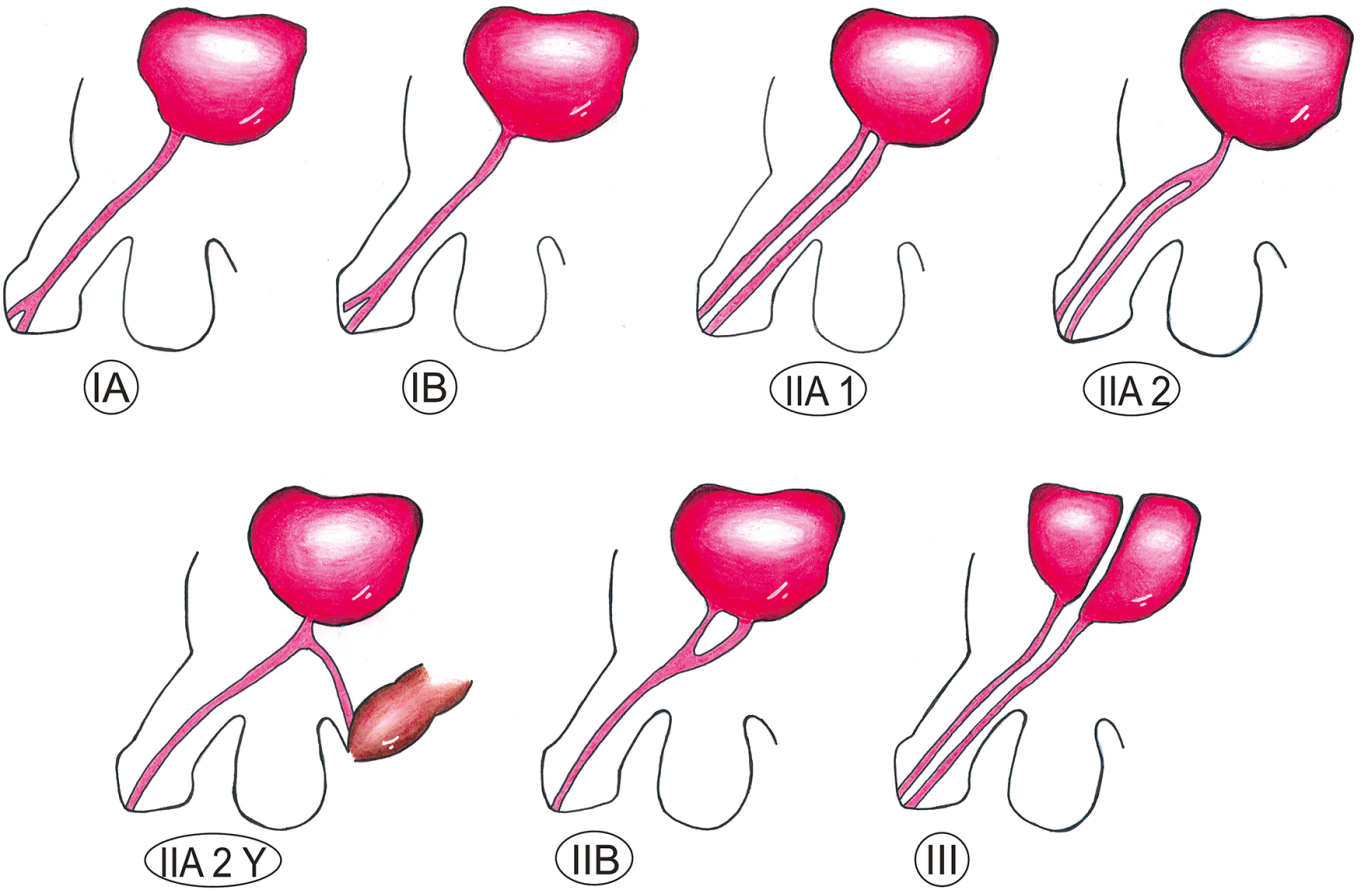
Effman classification of urethral duplications (4). IA: midline blind-ending channel on the dorsal or ventral surface of the penis, no connection to the bladder or urethra; IB: blind-ending tract originating from the urethra; IIA1: complete separate urethras originating separately from the bladder; IIA2: complete separate urethras with a common origin from the bladder; IIA2Y: the ventral urethra opens in the perineum; IIB: the urethras merge to form a single channel, which then opens up as a single meatus; III: complete duplication (bladder duplication), associated with caudal duplication syndrome.

The most widely used classification system for urethral duplication is the one proposed by Effman. This classification system is based on radiological findings (cystourethrography) and divides urethral duplications into incomplete (type I), complete (type II), and coronal (type III). This detailed classification of all aspects of the abnormality does not differentiate sagittal from coronal duplication (2, 3). Figure 1 shows the 7 most common types of urethral duplications—adapted from the original article (4).

Associated abnormalities are described in 25%–80% of different series and include duplication of the external genitalia or lower intestinal tract (most common in complete duplications), genitourinary abnormalities, spinal dysraphism, and midline disorders (5).

Treatment depends on the type of malformation and clinical presentation. Surgical intervention should be considered in symptomatic patients or for aesthetic reasons (6).

In the last two decades, we treated four patients with urethral duplications in our clinic. Two had prepubic sinus (7), and one boy had Y-type duplication. Several treatment options and their potential complications were evaluated during the planning stage of the procedures.

We conducted detailed research on urethral multiplicity and found that a comprehensive review of case reports and series can help clinicians better understand the range of manifestations and possible treatment options for patients with urethral multiplicity. By gaining insights into the various anatomical and functional presentations, clinicians can make more informed decisions based on higher evidence.

## 2 Methods

A systematic review has been conducted following the Preferred Reporting Items for Systematic Review and Meta-Analysis (PRISMA) methodological guide. A study protocol was published before conducting the review, and it has been registered in the international database of prospectively registered systematic reviews (PROSPERO ID CRD42023471685, https://www.crd.york.ac.uk/prospero/display_record.php?ID = CRD42023471685).

### 2.1 Search methods and sources

We choose the four most popular, widely available databases for this systemic review: PubMed®, PubMed Central® (PMC), Scopus, and Clarivate Analytics's Web of Science (WoS). The keywords we searched for were: “urethral duplication”, “urethral multiplicity”, “prepubic sinus” and “urethral triplication”. The search results were restricted to the last 15 years (2008–2023). The last search was done on 31/12/2023. All the results were downloaded in the corresponding format and uploaded to our account on the Covidence platform for screening and data extraction. All the required steps and tasks were followed. After removing the duplicates, title and abstract screening were done, followed by full-text screening by each reviewer.

### 2.2 Inclusion/exclusion criteria for study selection

Our review included all the case reports and case series (human studies) of urethral duplications, prepubic sinuses, and urethral triplications of pediatric-age boys (under the age of 18). We excluded studies presenting patients of adult age, female sex, with bladder duplication without urethral multiplicity, or patients with complete penile duplication and penile agenesis associated with urethral multiplicity. If the case series presented patients eligible for our inclusion criteria, we included them in our database, excluding only those not eligible participants.

### 2.3 Data extraction and synthesis

**Figure 2:**
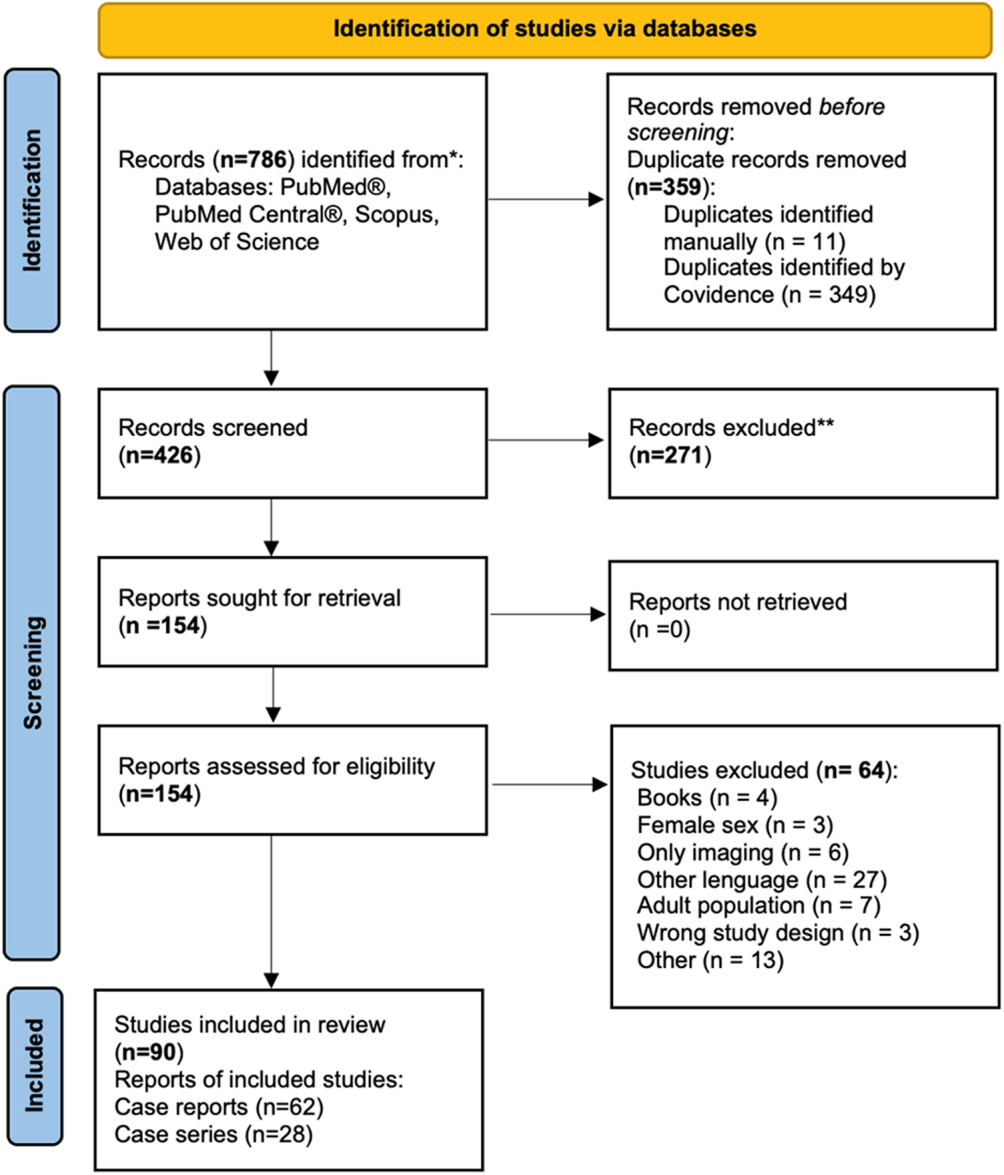
PRISMA flowchart of the screening process of the records.

We modified Covidence's template for data extraction to fit our preferences. As general information, we extracted from each paper the following (11 parameters): DOI number, Title, Lead author name, Continent, Country in which the study was conducted, Aim of study, Study design, Start date, End date, Study funding sources, Possible conflicts of interest for study authors. For participant(s) data, we noted (3 parameters): Inclusion criteria, Exclusion criteria, and Method of recruitment of participants. Our study aimed to obtain individual patient data of each participant from every included case report and case series in as much detail as possible. As baseline population characteristics, we extracted (22 parameters): Age at diagnosis, Age at intervention, Presentation, History of UTI (urinary tract infection)/obstruction, History of urinary incontinence, History (Other), Preop investigations (Genitourinary), Diagnosis made with, Description of Micturating cystourethrogram (MCU), Description of the urethra, Length, Other findings, Associated genitourinary anomalies, Other associated anomalies, Effman's classification, Surgical technique, Additional surgeries, Number of interventions, Complications, Follow up, Qol-questionnaires/Surveys, Outcome/Continence/Cosmesis, Histology, Number of excluded female patients. Data extraction was cross-checked independently by all four reviewers. Any disagreements were solved by consensus. The process is associated with a flowchart detailing the flow from the search through source selection, duplicates, retrievals, and any additions. The workflow is shown in Figure 2.

### 2.4 Quality assessment

Case reports and case series are uncontrolled study designs known for their increased risk of bias. Still, due to the lack of evidence, these publications on rare diseases such as urethral multiplicity have profoundly influenced the medical literature and continue to advance our knowledge (8). For quality assessment, we modified and individualized Covidence's Quality Assessment Template based on the JBI critical appraisal tool for case series (9), BMJ's methodological quality and synthesis of case series and case reports (8), and Joanna Briggs Institute's approach (10). One investigator carried out the assessment, and the others cross-checked it. In case of disagreement, it was resolved by consensus in the presence of the fourth reviewer. We searched for the following risks: (A) Were there clear criteria for inclusion in the case report/series? (B) Was the condition measured in a standard, reliable way for all the participants? (e.g., the study should clearly describe the method of measurement of the condition in a standard and reproducible way.) (C) Did the case series have consecutive inclusion of participants? (e.g., a consecutive inclusion is more reliable than those that do not: “We included all patients (x) with urethral duplication who presented to our clinic between 1992 and 2015” is more reliable than a study that states, “We report a case series of×patients with urethral duplication.”) /Does the patient(s) represent(s) the whole experience of the investigator (center), or is the selection method unclear to the extent that other patients with a similar presentation may not have been reported? (D) Was the patient's history clearly described and presented as a timeline? [A good case report will clearly describe the patient's history, including their medical, family, and psychosocial history, relevant genetic information, and relevant past interventions and outcomes. (CARE Checklist 2013)]; (E) Was/were the patient(s) presented clearly and adequately (e.g., demographics, existing health condition, physical and laboratory findings, and associated morbidities and Effman's classification for each patient separately)? (F) Were valid medical investigation methods used to identify the condition of all participants? (G) Were the details of the surgical treatment described adequately for each participant? (H) Were all the complications and their treatment (if possible) well described and reported? (I) Was the duration of the follow-up described for each patient separately? (J) Were the outcome results of the case/cases clearly described? Possible item ratings were yes, unclear, no, or not applicable. Data were extracted in “.xlt” format and analyzed further via Microsoft Excel. Descriptive statistics for variables were reported as median and min-max values, mean ± standard deviation (SD), absolute number (n), and percentage (%). The non-parametric Mann-Whitney U test was used to compare continuous variables.

## 3 Results

### 3.1 General characteristics

There were 90 studies included in the study. Of these, 62 were case presentations, and 28 were case series. The distribution of the studies across continents was as follows: Asia with 49, Europe with 16, North America with 9, South America with 4, Africa with 8, Australia with 1, and multi-continental 3 studies. The studies were conducted in 26 different countries, with India having the highest number of studies—20, followed by Turkey—12, and the United States—8.

### 3.2 Frequency

**Figure 3:**
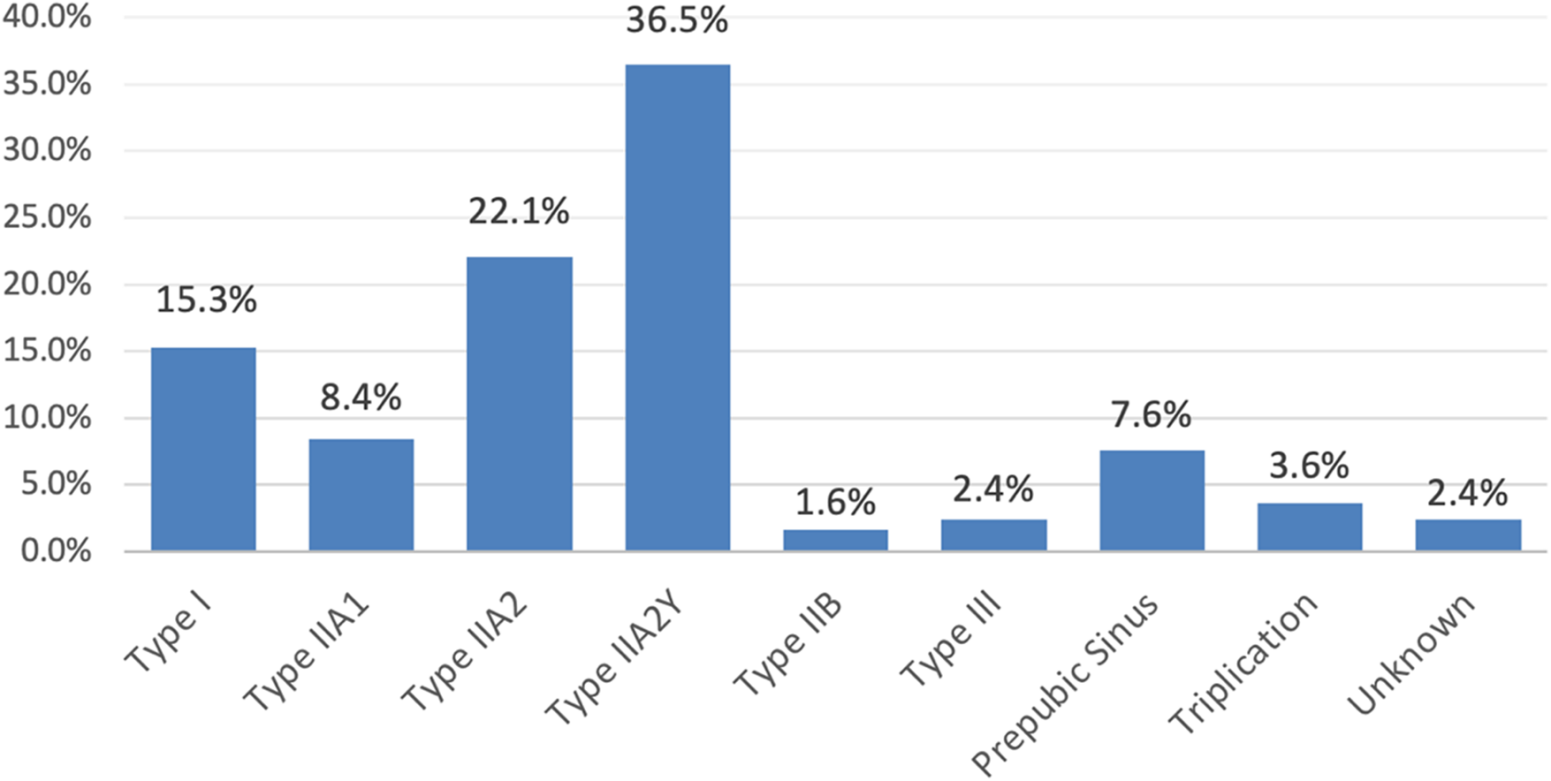
Percentile distribution of urethral multiplicity types.

The total number of patients included in our study was 250. Based on the classification of Effman, there were 38 cases of type I (15.3%), 21 cases of type IIA1 (8.4%), 55 cases of type IIA2 (22.1%), 91 cases of type IIA2Y (36.5%), 4 cases of type IIB (1.6%), and 9 cases of type III (2.4%). We also included 19 cases of prepubic sinuses (7.6%), 9 cases of triplication (3.6%), and 6 cases of unknown-type duplications (2.4%) in our review. Figure 3 illustrates the distributions of urethral multiplicity types.

### 3.3 Quality assessment

There were clear criteria for inclusion (97.77%), the conditions were measured in a standard, reliable way (90%), and there was a consecutive explicit inclusion and selection method (94.44%) for most of the included articles. The patient's medical history (63.33%), general presentation—including demographics, existing health condition, physical and laboratory findings, and associated morbidities and Effman's classification—(58.88%), complications (42.22%), follow-up (58.88%), and outcome (64.44%) were described adequately in approximately half of the papers. Also, we found qualitative information about the investigation methods (86.66%) and the applied surgical techniques (93.33%) in almost every report/ series.

The mean score value for all the included studies was 7.5 ± 1.91. Case series-type articles gained a lower score: 7.21 ± 2.2 vs. 7.62 ± 1.77 (*p* = 0.344). Also, there were more limited data, according to patient presentation, medical history, follow-up, and outcomes, in case series than in case presentations.

### 3.4 Prepubic sinuses

There were nineteen cases of prepubic sinuses. The age of the presentation was 4 years on average. In three cases, the diagnosis was made at birth, but the intervention was postponed for two years in two cases and three years in one case.

The most common symptom at presentation was discharge, reported in fourteen cases, that was purulent in seven instances, serous in three -, mucoid in two -, and unspecified in two cases. In two instances, erythema/inflammatory response (fever) was observed, and in one case, pain was reported. In terms of medical history, two instances involved circumcision, and two cases involved infection (pubic abscess). Treatment involved surgical intervention: complete excision in all the cases. Preoperatory investigations and the results of the histology exams are presented in Table 1. The outcomes were excellent in all the cases.

**Table 1:** Preoperatory investigations and histology of prepubic sinuses.

		Frequency
Investigations (19/19)	Ultrasound (abdominal or local)	11/19
	Fistulography/sinography: sinus tract with no communication with the (normal) bladder or urethra	10/19
	Micturating cystourethrography	7/19
	CT/MRI	5/19
	Cystoscopy, urethrocystoscopy	4/19
Histology (16/19)	Urothelium/transitional epithelium	7/19
	Squamous epithelium	5/19
	Both urothelium and squamous epithelium	4/19
	Inflamed fibrous tissue	4/19

### 3.5 Type I urethral duplications

Thirty-eight cases of type I urethral duplications were identified, including type IA in twenty-two and IB in eight cases, according to the Effman classification and not specified in eight cases. The average age at the time of intervention was 4 years and 7 months, and in six cases, the intervention was postponed or delayed.

A visible secondary meatus was observed at presentation in twenty-seven out of thirty-eight cases. This meatus was located dorsally/epispadiac/peno-pubic positions in sixteen cases and ventrally/proximally/hypospadiac/peno-scrotally in seven cases. Eight patients didn't experience any symptoms (the malformation remained asymptomatic), while six boys had discharge that was either sero-mucoid or purulent in 3–3 cases. Other symptoms included double urinary stream, infection in 2–2 cases, and dorsal chordee and distal hypospadias in 1–1 cases. Five boys had a history of circumcision, while five others had infections such as UTI or penoscrotal abscess. The preoperative investigations, associated anomalies, and treatment options in type I urethral duplications are listed in Table 2.

**Table 2:** Preoperative investigations, associated anomalies, and surgical technique options for type I urethral duplications.

			Frequency
Investigations	Micturating cystourethrography		15/38
	Ultrasound (abdominal or local)		15/38
	Cystoscopy/Urethrocystoscopy		9/38
	Retrograde urethrogram		9/38
	Urethrography		4/38
	Probe insertion		1/38
	Penile sinogram		1/38
Associated anomalies 15/38	Genital	Chordee	6/38
		Preputial deficiency	5/38
		Webbed penis	1/38
		Cleft of the glans	1/38
	Urinary	VUR	2/38
		Hypospadias	2/38
		Multicystic kidney	1/38
		Solitary kidney	1/38
		Urethral diverticulum	1/38
	Systemic	Down syndrome	1/38
		ARM	1/38
		Pubic diasthasis	1/38
Surgical technique options	Complete excision of the accessory urethra		15/38
	Urethro-urethrostomy		5/38
	Excision of the accessory urethra via stripping		3/38
	Urethroplasty		3/38
	Endoscopic diatermocoagulation		1/38
	No intervention		9/38

VUR, vesicoureteral reflux; ARM, anorectal malformation.

In addition to the surgical treatments listed in Table 2, various other procedures were performed. Two cases underwent chordectomy, two cases received glanduloplasty/penoplasty, circumcision was carried out in three cases, TIP repair in one case, Browne hypospadias repair in one case, incision of the web in one case, and diverticulectomy in one case. All cases had excellent outcomes without reported complications, and follow-up was conducted for up to six years. Histological examination was only presented in three cases, which revealed urothelium in two cases and stratified keratinizing epithelium in one case.

### 3.6 Type IIA1 urethral duplications

There were a total of twenty-one cases of type IIA1 urethral duplications, with an average age of presentation being 3 years and 6 months. Out of these cases, eleven were diagnosed at birth.

The most common presentation was a visible secondary urethral opening, with sixteen cases reported. Among these cases, eleven were dorsal/epispadiac, and one was hypospadiac. Other presentations included incontinence (six cases), two urinary streams (two cases), swelling during voiding (one case), poor flow (one case), recurrent UTI (three cases), bladder exstrophy (three cases), and fecaluria (one case).

Regarding functionality, nine out of the twenty-one cases had a normal functional apical urethra, while three cases had an incontinent epispadiac/dorsal urethra. Two cases had a main hypospadiac meatus, and two had urine dribbling from the penile urethral meatus and passed urine in a stream from the anterior anal verge. Two boys had anal urinary leakage.

Three boys had a history of circumcision, and three cases had a history of recurrent UTIs.

Preoperative investigations, associated anomalies, and treatment options are listed in Table 3.

**Table 3:** Preoperative investigations, associated anomalies, and surgical technique options for type IIA1 urethral duplications.

			Frequency
Investigations	Micturating cystourethrography		15/21
	Ultrasound (abdominal or local)		13/21
	Cystoscopy, urethrocystoscopy		11/21
	Retrograde urethrogram		4/21
	Pelvic x-ray		2/21
	Intravenous pyelography		1/21
	Dye study		1/21
	CT		1/21
	Renal scintigraphy		1/21
Associated anomalies 14/21	Genital	Chordee	2/21
		Preputial deficiency	1/21
		Testicular atrophy	1/21
	Urinary	Renal: crossed ectopy, dysplasia, solitary -, horseshoe kidney	4/21
		Bladder: exstrophy, duplication of the neck, septum	4/21
		VUR	2/21
		Megalourethra	1/21
		Hypospadias	1/21
	Systemic	Abdominal wall defect	1/21
		ARM	1/21
		Pubic diasthasis	1/21
		Anal incontinance	1/21
		Sigmoid and rectum dupplication	1/21
		Vertebral	2/21
		Neurologic	1/21
Surgical technique options	Complete excision of the dorsal/epispadic urethra		7/21
	Complete penile disassembly		4/21
	Urethro-urethrostomy		4/21
	Staged techniques (three/two)		2/21
	Hypospadias repair only		1/21

In fourteen cases, the duplications were corrected in a single-stage manner. However, there were also cases where up to 5 interventions were required. Complications were presented in five cases, which included fistula formations in three, dorsal penile skin loss, and stenosis in 1–1 cases. The follow-up period ranged from 9 months to 14 years, and the outcomes were functionally and aesthetically satisfactory.

### 3.7 Type IIA2 urethral duplications

There were fifty-five cases of type IIA2 urethral duplication that presented at the age of 3 years on average. The presentation was related to morphology and function symptoms. Morphology-related symptoms included a visible secondary meatus in fifteen cases, with two being orthotopic, four being epispadiac, one being hypospadiac, one having bladder exstrophy, and one having splitting. Function-related issues included a double urinary stream in twelve cases, with three having a more functional ventral meatus and one having both functional meatuses. Other function-related issues included incontinence in eight cases, straining in four cases, thin stream in two cases, swelling of the penis increasing with urination in one case, flow from the umbilical region in one case, no voiding in one case, painful terminal hematuria in one case, and asymptomatic in four cases.

The history of these cases included UTI in thirteen cases, circumcision in five cases, colostomy in three cases, prematurity, operated Fallot tetralogy, and orchiectomy for teratoma in 1–1–1 cases.

Preoperative investigations, associated anomalies, and treatment options are listed in Table 4.

**Table 4:** Preoperative investigations, associated anomalies, and surgical technique options for type IIA2 urethral duplications.

			Frequency
Investigations	Micturating cystourethrography		28/55
	Cystoscopy, urethrocystoscopy		25/55
	Ultrasound (abdominal or local)		20/55
	Retrograde urethrogram		16/55
	Intravenous urethrogram		4/55
	MRI		3/55
	Urodynamic exam		1/55
	Dimercaptosuccinic acid scan		1/55
	Renal scintigraphy		1/55
Associated anomalies 35/55	Genital	Peno-scrotal: transposition, web, bifid scrotum	5/55
		Meatal stenosis	3/55
		Irregular/hooded foreskin/phimosis	3/55
		Chordee	1/55
		Testicular teratoma	1/55
		Micropenis	1/55
	Urinary	Vesicoureteral reflux	12/55
		Renal: crossed ectopy, dysplasia, solitary -, horseshoe kidney	10/55
		Ureteral: ectopic posterior urethral insertion, duplication, megaureter	6/55
		Bladder: diverticle, neurogenic	2/55
		Urethal: diverticle, stenosis, posterior urethral valves	7/55
		Prostatic utricule cyst	1/55
		Patent urachus	1/55
	Systemic	Spina bifida, lipomyelocele, lipoma filum terminale	7/55
		Tethered cord	5/55
		Anorectal malformation	6/55
		Vertebral	2/55
		Hirschsprung	1/55
		VACTERL	1/55
		Klinefelter syndr.	1/55
Surgical technique options	Complete excision of the accessory urethra		22/55
	Urethro-urethrostomy (1-stage/staged)		8/55
	Urethroplasty/meatoplasty alone		7/55
	Complete penile disassembly (Mitchell-Caione)		2/55
	Vesicostomy/urethrostomy/bladder augmentation/Monti/Mitrofanoff		10/55

Regarding contrast studies, in fourteen cases, a single channel arising from the bladder was described, splitting into two, coursing independently to the meatuses.

The dorsal tract is usually less developed and stenotic at its origin, while the ventral tract can present dilations, diverticula, and tortuosities at the base and can be stenotic distally. In some cases, both urethras are functional and of good caliber.

Outcomes are generally good. However, stress incontinence, dysuria, straining, psychological problems, and erectile dysfunctions have been reported. Complications were described in nine cases, with eight cases of stenosis (successfully treated with dilatations in five and requiring Mitrofanoff in three cases). Follow-up was conducted for up to 12 years.

### 3.8 Type IIA2Y urethral duplications

There were a total of ninety-one cases of type IIA2Y urethral duplications.

In this specific type of urethral duplication, twenty-five patients were described as having a predominantly ventral type of voiding. Seven patients presented with a double urinary stream; in six cases, the micturition was primarily orthotopic (dorsal), whereas four patients voided only per rectum. Morphologically, ten patients were observed to have two urethral orifices, one orthotopic or epispadiac (normal sized, or as a blind-ending megalourethra, or as a small orifice), and a ventral opening in the rectum, perineal region, raphe, or at the penoscrotal junction.

There were several complaints related to function, including dribbling from various areas (ten cases) such as the prepuce (one case), glans (two cases), near the dentate line (one case), anus (four cases), and perineum (two cases); voiding with two streams (seven cases); voiding per rectum since birth (five cases); incontinence (three cases), dysuria (two cases), straining (one case), inappropriate bladder drainage (one case), wetting the perineum (one case), and sitting on the toilet when urinating (one case). Additionally, there were eight cases of UTIs, one case of abdominal distension, and one case where catheterization was impossible.

The orthotopic urethra was either stenotic, atretic, or hypoplastic in forty-two cases; it was partially stenotic in thirty cases and was of good caliber in nineteen cases. The length of the stenotic part varied from case to case. In sixty-six cases, the ventral urethra was in an anal or rectal position, in eighteen cases, it was in a perineal position, three cases were abortive, and in four cases, it was of an unspecified position.

The perineal opening of the ventral urethra is not always on the midline. It can be found in the gluteal area at either 3 or 11 o'clock. The ventral urethra had a better caliber. However, eight patients had stenotic ventral urethra. When both urethras are stenotic or abortive, renal failure is the consequence.

Preoperative investigations, associated anomalies, and treatment options are listed in Table 5.

**Table 5:** Preoperative investigations, associated anomalies, and surgical technique options for type IIA2Y urethral duplications.

				Frequency
Investigations	Micturating cystourethrography			42/91
	Cystoscopy, urethrocystoscopy			35/91
	Ultrasound (abdominal or local)			39/91
	Retrograde urethrogram			31/91
	Intravenous urethrogram/pyelogram			15/91
	Urethrocystrogram			7/91
	MRI Urography/CT			5/91
	Endoscopy			4/91
	Colostogram, Ano/rectoscopy			3/91
	Renal scintigraphy			2/91
Associated anomalies 40/91	Genital	Peno-scrotal: transposition, bifid scrotum		8/91
		Dysplastic corporea cavernosa		2/91
		Irregular foreskin		1/91
		Seminal vesicle reflux		1/91
	Urinary	Vesicoureteral reflux		13/91
		Renal: hypoplasia, dysplasia, multicystic, duplex, solitary -, horseshoe kidney		13/91
		Ureteral: duplicity, ectopic orifice, megaureter, obstructive megaureter, hydroureteronephrosis		7/91
		Bladder related		2/91
		Urethal: cyst, stenosis		7/91
	Systemic	Digestive	Anorectal malformation	7/91
			Oesophageal atresia	5/91
			Duodenal atresia	6/91
			Malrotation	2/91
		Cardiac	Double-outlet right ventricle	1/91
			Tetralogy of Fallot	1/91
			ASD, PDA	1/91
		Vertebral		5/91
		Other	Sezuires	1/91
			VACTERL	2/91
Surgical technique options	Urethro-urethrostomy			19/91
	Complete excision of the ventral urethra			14/91
	Staged procedures		5 stage	1/91
			4 stage	4/91
			3 stage	7/91
			2 stage	17/91
	Urethroplasty		BMGT	14/91
			Preputial tube	14/91
			Q flap	6/91
			TIPF-DD	3/91
			Duckett	1/91
			Mathieu	1/91
			Snodgrass	1/91
			Bracka	1/91
			Thiersch- Duplay	1/91
			Tunica vaginalis flap	1/91
	Initial vesicostomy/perineal urethrostomy			10/91
	Anterior sagittal anorectoplasty/ASTRA			9/91
	Mitrofanoff/Monti			8/91
	PADUA/ dilatations			6/91

TIPF-DD, transverse inner preputial island flap based on dorsal penile dartos flap; BMGT, Buccal mucosa graft tabularization.

Complications that were observed during follow-up for up to 30 years included meatal stenosis, recalcitrant stenosis, multiple stenosis, anastomosis site stenosis (nineteen cases), fistula (seven cases), infection (three cases), urethral diverticulum (one case), stenosis that required Mitrofanoff (one case), fibrosis of the neourethra (two cases), wound dehiscence (one case), and urinary catheter malfunction (one case).

The outcome was variable.

### 3.9 Type IIB urethral duplications

There were only four patients presenting this rare type of urethral duplication. They presented, on average, at the age of 7 years.

The patients complained about various symptoms such as straining or had neuropathic bladder while urinating with single-stream. The diagnosis was made incidentally.

Preoperative investigation showed that two patients had undergone MCU and RU, while two others had undergone cystourethroscopy and ultrasonography.

Associated anomalies were also found in these patients, such as a scrotal abscess in one, a non-functional kidney in another, and caudal regression and a tethered cord.

The surgical techniques used for these patients included urethra-urethrostomy, excision of the accessory urethra, and dilatation of the urethra—in case of stenosis.

### 3.10 Type III urethral duplications

There were six patients with an average age of 4 years. Their presentation included visible secondary meatus, with glandular, penile, perineal, and recurrent UTIs being the most common. Preoperative investigations included methylene blue injection, cystoscopy, fluoroscopy, ultrasound, micturating cystourethrography, MRI, CT, retrograde urethrography, and intravenous urography. Associated anomalies were observed in the genital, urinary, and general areas.

Surgical interventions included mobilization of urethras, side-to-side urethrostomy, penoscrotal urethrostomy, excision of the dorsal accessory urethra, and no treatment. No complications were reported, and follow-ups were done for up to one year.

### 3.11 Urethral triplication

There were a total of nine patients with various symptoms: three of them urinated from the rectum, one had drops from the epispadic urethral meatus, one had abnormal foreskin, one had perineal voiding, one had enlargement of the penis during voiding, one had dribbling, and two had a double urinary stream. Additionally, one patient had a fever, one had abdominal pain, one had visible accessory urethras, one experienced fecaluria, one had incontinence, one had UTI, and one had dysuria.

**Figure 4:**
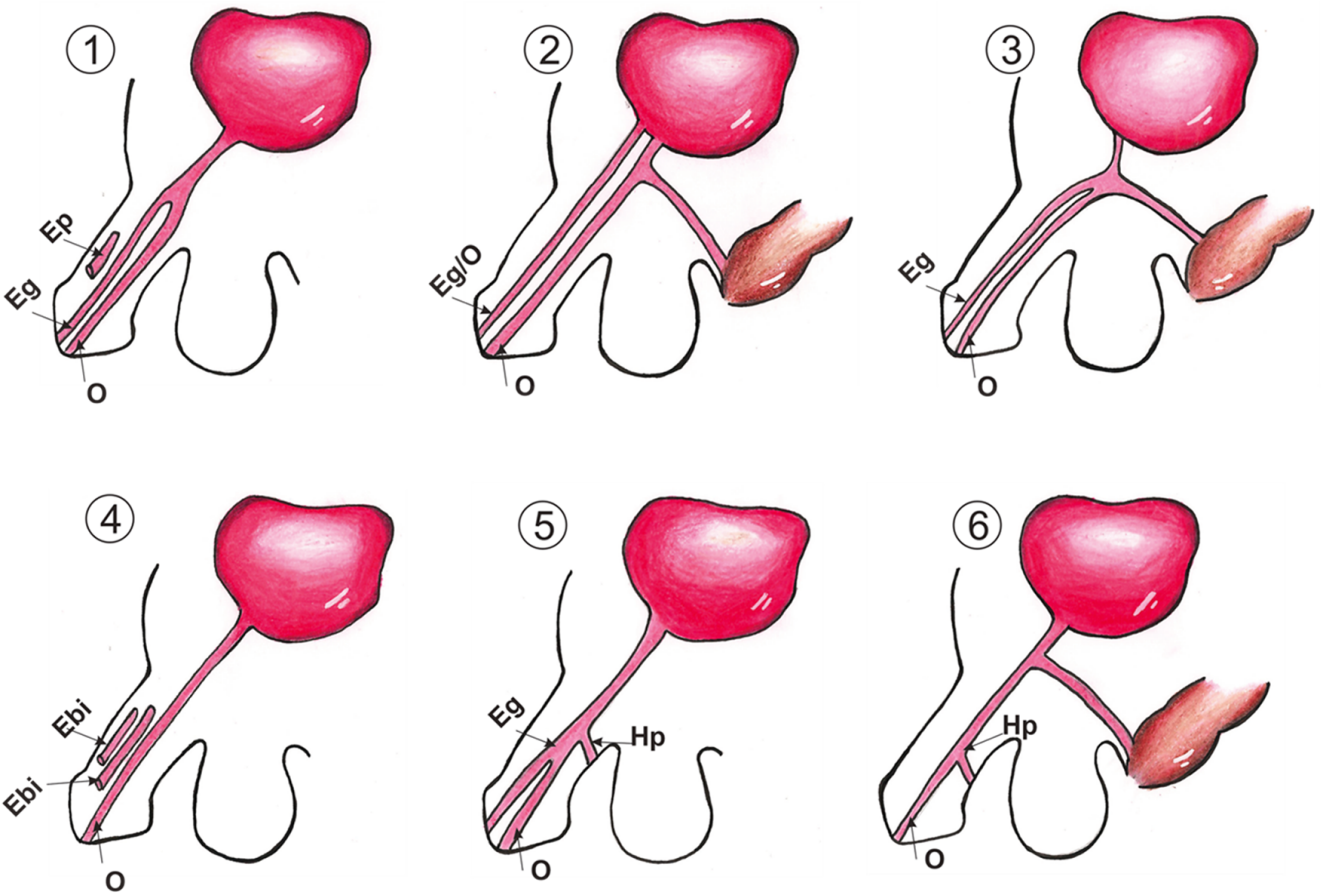
Anatomical variants of urethral triplications. Eg, epispadiac glandular meatus; Ep, epispadiac penile meatus; O, orthotopic meatus; Hp, hypospadiac penile meatus.

There are some possible anatomical variations. There is always an orthotopic functional urethra with one or two epispadiac urethras, which open glandularly or on the dorsal surface of the penis. Ventrally situated urethras might open in the rectum/anus/perineum or on the ventral surface of the penis. In some cases, there are only epispadiac or only hypospadiac secondary urethras. Figure 4 represents the variations of urethral triplications we found. We encountered two of each 1,2,3 variations and one of each 4,5,6. As a particularity, four cases showed a Y triplication (11–14): a common proximal urethra divided into three channels.

Preoperative investigations, associated anomalies, and treatment options are listed in Table 6.

**Table 6:** Preoperative investigations, associated anomalies, and surgical technique options for urethral triplications.

			Frequency
Investigations	Micturating cystourethrography		7/9
	Cystoscopy, urethrocystoscopy		5/9
	MRI/CT		3/9
	Anterior/retrograde UG		2/9
	IVU		1/9
	Catheterization		1/9
Associated anomalies 7/9	Genital	Hypospadias	1/9
		Penoscrotal transposition	1/9
		Chordee	1/9
	Urinary	Hydronephrosis/ VUR	2/9
		Renal: dysplasia, multicystic, duplex, solitary -, horseshoe kidney	4/9
		Bladder septum	1/9
	Systemic	Cerebral palsy and hypotonia, tethered cord, lipoma filum terminale	3/9
		Vertebral	2/9
		Duplicated sigmoid, rectum, appendix	1/9
Surgical technique options	Perineal removal of the accessory urethra		5/9
	Excision of the epispadiac urethra		2/9
	Urethrostomy of the 3 urethras		2/9

There were no reported complications. Follow-up: up to 4 years. The outcome was satisfactory in most of the cases.

## 4 Discussions

Urethral multiplicities are rare abnormalities that most pediatric surgeons and urologists do not encounter frequently. There are no dedicated textbook chapters or systematic literature reviews on this condition, and the available information is limited to case reports and case series. These are uncontrolled study designs known for their increased risk of bias. Still, without stronger evidence, data from these case presentations and series might profoundly influence the medical literature and contribute to advancing our knowledge (8).

Approximately 300 cases of urethral duplications have been reported in the literature; articles mainly refer to this number (15). As a main goal, we determined the exact number based on the published data. We also identified other forms of urethral multiplicities that might be included in this group of malformations due to their embryological and morphopathological origins and similarities. These are the prepubic sinuses and the urethral triplications. These malformations have never been discussed together in the literature.

Similarly to the real number of existing cases, the distribution of the different types of urethral multiplicities and duplications hasn't been described yet. Our study defines the anatomy and frequency of each type of multiplicity and sheds light on the distributions of the different anatomical variants.

The incomplete forms of urethral duplications represent almost a quartile of cases. These are the type I duplications. Prepubic sinuses might also be part of this group. Type IIAs are the most common forms of duplication, representing over two-thirds of all cases. Among the IIA types, IIA1 is the least frequent. Surprisingly, the most frequent type is the IIA2Y type, representing over a third of all cases of multiplicities. This type is also the most challenging and the most spectacular form. The type IIB, III, and triplications are rare. Complex cases were also described which do not fit perfectly into a single category.

Prepubic sinuses are tracts situated dorsally to the main urethra. They have an orifice opening distally and usually blind ending proximally. Recent theories suggest that prepubic sinuses may be a form of urethral duplication despite different etiological concerns. Immunohistochemical studies showed the presence of transitional epithelial cells in prepubic sinuses (16). Our findings also support this suggestion: in almost all cases, the excised specimens showed the presence of urethral tissue. Stephens also described the prepubic sinus as a subgroup of urethral duplications (17). He differentiated three subtypes: subtype 1, as a parallel dorsal channel (complete or incomplete) to the urethra; subtype 2, as an epispadiac channel from the dorsum of the penis to the bladder or urethra; and subtype 3, as a dermoid sinus from the dorsal base of the penis, toward the dorsum of the pubic symphysis or to umbilicus (18–21). Another classification describes the prepubic sinuses as high type, with the trajectory extending to the urachal remnant; middle type, with the trajectory extending to the bladder; and the low type, with the trajectory extending to the urethra (22).

We found a consensus regarding the treatment: complete sinus excision. In all articles, the procedure consists of open surgery. In one case, there was described an endoscopic/laparoscopic preperitoneal (extraperitoneal) approach for a high sinus with suspicion of bladder communication (infirmed) (22). The outcome was good in all cases, with no reported complications.

In the case of type I urethral duplication the accessory urethra is usually dorsally found, but there are also ventral or hypospadiac forms. A boy had a collateral partially duplicated urethra with congenital anterior urethral diverticulum (23). All dorsal accessory urethras had small dimensions. In one case, it was only a dorsal median fissure (24), in another—incomplete IB type—a blind channel at both ends (25).

The most common surgical technique used to repair type I duplications involves the removal of the accessory urethra. We believe this approach is the most logical for dorsal, atretic accessory urethras, which represent most cases. The alternatives—less invasive—were the stripping of the urethra (26) or endoscopic diatermocoagulation (5). A urethroplasty was performed for the ventral accessory urethra—for example, to a patient with orthotopic, atretic urethra, and ventral hypospadiac urethra (27, 28). A variant was urethra-urethrostomy via a septal incision (crushing and division of the common septum) (28). Urethroplasty was the solution also for the urethral diverticulum. The outcome was good in all cases, with no complications.

Type IIA1 urethral duplication is a rare condition. All these duplications were in a sagittal plan except one in a coronal plan (29). In all the cases, the ventral urethra was the functional one. The dorsal urethra could be of normal caliber (30), stenotic (31), incontinent (32), or dilated as a megalourethra (33).

In more than half of the cases, it was an obvious epispadiac or dorsal accessory urethra. Unusual presentations might include a glandular or hypospadiac and continent good caliber urethra; or a ventral urethra opening on the anal verge with anal urinary discharge. They resemble IIA2Y type duplication, which is more frequent, but in that type, the urethras emerge from the bladder by a single common channel. It has been proposed that a specific name be given to this individual form: type IIA3 (31). Also, epispadiac or dorsal urethral plate malformations were described as continued with bladder exstrophy (26, 34). An interesting case included coronal duplication, with the two orifices side-by-side and the right urethra connected with the rectum, manifesting with fecaluria. This malformation was associated with cataracts, sigmoid duplication, and bladder septum (29).

Each child was operated using individualized techniques. The most manageable situation was when a dorsal accessory urethra was found, and a ventral orthotopic urethra was present. The excision of the dorsal accessory urethra was an option—as proximal to the bladder (next to the bladder or behind the pubic symphysis) (26, 28, 35–38). In more than half of the cases, the total or partial excision of the dorsal urethra was performed for different reasons. Caione et al. presented their procedure: complete penile disassembly with the total removal of the dorsal accessory up to the bladder neck (36). In other cases, urethro-urethrostomy was performed (6, 33, 39). The two urethras were connected, and the urethroplasty was made to complete the operation. Two patients remained with the two openings, having no functional problems (29, 30). Patients with anal ventral urethra needed complex operations: vesicostomy or colostomy, perineal urethrostomy, and staged urethroplasty to advance the urethral opening as possible to the tip of the glans (31, 40). They reported between three and six procedures to accomplish correct the anomalies.

We have to mention that the articles included for the IIA2 urethral duplication group we found dorsally placed (epispadiac) and ventrally placed (hypospadiac) accessory urethras. The ventral urethras were more functional. Proximally dilated and distally atretic (41) and ventral atretic urethra, which needed excision (37), were described.

Excision was the most used technique in this group. The accessory urethra was prepared and dissected as proximal as possible, ligated at the base, and excised. As an alternative to excision, it was described as a cauterization of the accessory dorsal urethra (42). Another procedure was urethra-urethrostomy: the two urethras were connected in an end-to-end or side-to-side fashion. For example, in these cases, in the proximal part, the ventral urethra had a better caliber. It was anastomosed with the dorsal orthotopic urethra, which had an acceptable caliber in the distal part (43, 44). Another procedure was the urethroplasty—usually when the dorsal urethra was stenotic (and could not be utilized) and the ventral one was hypospadiac (26, 35, 40). Various techniques used for hypospadias repair with flaps and grafts and staged procedures were described.

In most cases, good results are claimed. The urethroplasty was also utilized to reshape a urethra dilatation (1). Dilatations were performed classically or via the PADUA procedure (35). There were some complex cases in which the reconstruction of at least one urethra failed, and the patient had to be referred to an extreme procedure such as Monti or Mitrofanoff (40, 45). In some cases, we must note that temporary vesicostomy was utilized as a stage for the final reconstruction of the urethra.

A variant of type IIA2 is the IIA2Y type or Lambda (*λ*) urethral duplication (46). It represents the most debated and the most controversial type of urethral duplication.

The literature has not agreed on an appropriate terminology for this condition (47). Firstly, the pathology was mentioned as a congenital “H type” anourethral fistula—like the tracheoesophageal fistulas (48). In Bates and Lebowitz's opinion, Y-type duplication might represent two different pathologies: “urethral duplication”, when the patient urinates mostly through the anus with hypoplastic penile urethra, and “urethral fistula congenital”, when the functional penile urethra (49).

Our study showed that IIA2Y is the most frequent type of urethral duplication. The popularity could be due to its spectacular morphology and the therapeutic challenges it represents. We found twenty-seven articles about this malformation. There are single-case presentations and some large case series up to seventeen cases (50).

When it comes to anatomy, the quality of the orthotopic urethra is the most crucial factor to consider when trying to restore it. We found that it could be stenotic, atretic, or hypoplastic—in nearly half of the cases, partially stenotic in a third, and had a good caliber in over one-sixth. Partially stenotic means that the proximal part is stenotic, and the distal part has a normal diameter. On this line, Kurian, in a large case series, proposes to subtype the Y duplications into four types: type l with orthotopic urethra completely stenotic, type II with orthotopic urethra partial (proximal) stenotic, type III resembling type II, but the proximal stenotic segment is shortened, type IV with orthopic urethra having a normal caliber (50). Lima proposes three groups of anatomy: type A with both urethras having good caliber, type B with orthopic urethra stenotic and good ventral urethra, and type C with both urethras abortive (46). This dorsal urethra can be dilated in the distal portion, forming a megalourethra.

In most cases, the ventral urethra is in the anal or rectal position, also perineal and abortive verges were described. Perineal openings are not always on the midline.

The choice of surgical technique is influenced by various factors such as the patient's anatomy, the surgeon's skill level, complications, or associated abnormalities.

The complete excision of the ventral urethra could be done when the orthotopic urethra had a good diameter or could be successfully dilated.

Different types of urethroplasties were performed, and usually, the reconstructions required multi-stage urethroplasties. ASTRA, Snodgrass, Q–flap, Mathieu, Bracka, on-lay flaps, or buccal mucosa graft urethroplasties were described. Usually, in the first step, the ventral urethra is detached from the anus and brought into a perineal position with the reconstruction of the anal region. The urethra could be reconstructed from the shaft of the penis or the perineum to the glans. A lot of complications appeared during these plasties. Only a single case series reported good results in all patients (six) using a long so-called “Q-flap” urethroplasty (51).

A urethral-urethra anastomosis could also be performed. After the dissection of the ventral urethra, it is brought into a hypospadiac position and then anastomosed with the distal non-stenotic part of the orthotopic urethra. In this way, the Y duplication is transformed into an IIB. This procedure is also prone to many complications.

The perineal urethrostomy is another described possibility. When the functional urethra is positioned in such a way that it is compressed by the anal sphincter, bringing it to the perineal position helps to maintain proper urine output and prevent obstruction and infections. After many complications, Macedo et al. affirmed: “actual tendency, as a definitive strategy, was to leave the perineal stoma” (52).

Monti or Mitrofanoff procedures were made in patients with important complications or completely stenotic orthotopic urethra or abortive both urethras.

Simple dilatation of the orthotopic urethra was also tried in one case, and there were no interventions in two cases.

We only found three articles about IIB urethral duplication. Due to this particularity and the lack of its symptoms, we think most IIB duplications remain silent and undiagnosed. Diagnosis may be incidental or due to associated anomalies. Treatment is only required in symptomatic cases. In one case, a urethro-urethrostomy was performed; the patient developed a stricture, and a dilatation was performed (35). In another boy, just dilatation of the ventral urethra was made. A boy who developed a scrotal abscess needed the excision of the accessory urethra (53), and a boy with a neuropathic bladder had no surgical intervention (54). All those patients were doing well with no complications.

Type III duplications may be associated with a caudal duplication. In most cases, the ventral urethra was the more functional one. The dorsal urethra was permeable in half of the cases. In one case, it was specified that the accessory bladder was anteriorly situated, reporting to the normal bladder (28). There was a case where both urethral orifices were situated perineally and coronally. The urethras were mobilized, brought to the basis of the penis, and anastomosed side-to-side as a “shotgun” in a hypospadiac position, waiting for a urethroplasty (55). The case also presented here had two bladders and two urethras united in a single channel. No intervention was done. Many patients had no urinary complaints and were incidentally diagnosed, requiring no treatment.

A triplication is a rare finding; we discovered only a few cases. The anatomy is diverse, and the treatment and clinical manifestations must be adapted. In one case with both epispadiac accessory urethras, the most epispadiac urethra was excised (56). In another case, both accessory urethras were openly excised, and an accessory anterior bladder was excised using a robotic approach (57). In half of the cases, the anal urethra was excised, having the advantage of a normal orthotopic urethra. In other cases, the third urethra was anastomosed, end-to-side with the orthotopic urethra (1, 12). Urethro-urethrostomies were also performed within the main urethra anastomosed with the epispadiac accessory urethra end-to-side (1) or fused to form a single urethra (12). If the second urethra was in a hypospadiac position, a urethroplasty was performed between that urethra and the main urethra after the excision of the rectal urethra (13). No therapy was also reported (14).

In summary, we have identified multiple effective therapeutic options for urethral duplications and multiplicities that will help in making an informed decision:

- Doing nothing if the patient has no clinical complaints and the malformation is not evident.
- Excision of the accessory urethra (usually dorsal but rarely can be ventral) if the orthotopic urethra has a good caliber or can be dilated enough.
- Urethro-urethral anastomosis (end-to-end or side-to-side)—when the orthotopic urethra is stenotic in the proximal segment, and the accessory urethra (usually the ventral) has a better caliber. This procedure transforms a IIA1, IIA2, or type or Y type duplication into something like type IIB duplication, which is a better situation. Urethro-urethrostomy between the two (parallel) urethras might also be possible. This procedure may be completed with a urethroplasty.
- Urethroplasty—when the problem mainly affects the distal part of the urethras. Different types of urethroplasties were proposed with heterogenous results. Usually, these are multi-stage procedures.
- Dilatation of the orthotopic urethra, followed or not with the excision of the accessory urethra for the stenotic orthotopic forms.
- Perineostomy for complicated different IIA types.
- Initial Mitrofanoff or Monti appendico-/illeovesicostomy in the very complicated types or cases of irremediable stenosis/obstruction.

## 5 Conclusions

Urethral multiplicities are rare and heterogenic group of malformations. Clinical manifestations can vary from silent and atypical to very specific complaints. For successful management, performing a good quality imagistic examination is a must. The treatment is specific to each patient and for each morphological type and may even depend on the surgeon's preference or skill. Our review analyzed the individual data of patients with urethral multiplicities from the last fifteen years’ case presentations and series. This could serve as a resource for pediatric urologists and specialists seeking to enhance their knowledge of the field, with the aim of providing superior care to their patients.

## Data Availability

The original contributions presented in the study are included in the article/Supplementary Material, further inquiries can be directed to the corresponding author.
