# Supplementary material for: Urethral multiplicity in boys: systematic review of case reports and case series from the last 15 years
Source: Front Pediatr. 2024 Jun 11;12:1404947. doi: 10.3389/fped.2024.1404947 (PMC11196844; doi:10.3389/fped.2024.1404947)
Supplement: Supplementary file 1 [file Datasheet1.pdf]

Urethral multiplicity in boys: Systematic review of case reports and case series from the last 15 years

Supplementary material
List of the included studies

1. Eroglu E, Ozen MA, Gundogdu G. Congenital prepubic sinus. Arch Dis Child (2016) 101:585. doi: 10.1136/archdischild-2015-310205
2. Kim HY, Shin OR, Jang ED, Yoon BI, Chung MS, Chung YS, Han CH. Congenital prepubic sinus: A variant of urethral duplication. Korean J Urol (2007) 48:881–884. doi: 10.4111/kju.2007.48.8.881
3. Nonnenmacher G, Rhein D, Loff S. Congenital prepubic sinus: Rare diagnosis with unclear etiology. J Pediatr Surg Case Rep (2016) 9:16–18. doi: 10.1016/j.epsc.2016.04.001
4. Sinha AK, Kumar B, Kumar P, Kumar M, Kumar A, Sahay N. Congenital pre-pubic sinus: An embryological enigma. J Pediatr Surg Case Rep (2017) 18:36–38. doi: 10.1016/j.epsc.2016.12.003
5. J AM, M BA, L AG, Hernández-Martín S, Montes M, R GT, C BP. Congenital Prepubic Sinus as a Variant of Incomplete Urethral Dorsal Duplication: A Case Report with New Insights into its Immunohistochemical Characterization and a Comprehensive Literature Review. Int J Surg Pathol (2023) 31:680–688. doi: 10.1177/10668969221117239
6. Wang C, Ma X. Congenital prepubic sinus with dorsal penile curvature: a case report and literature review. BMC Pediatr (2019) 19:367. doi: 10.1186/s12887-019-1768-0
7. Nasir AA, Abdur-Rahman LO, Olaoye I, Oyinloye AO, Bamigbola KT, Adeniran JO. Congenital prepubic sinus: a variant of epispadiac dorsal urethral duplication. J Pediatr Urol (2013) 9:e82-5. doi: 10.1016/j.jpurol.2012.09.010
8. Ozdemir E, Yildiz T, Kanbay M, Kanbay S. A case of congenital prepubic sinus. Eur J Pediatr Surg (2011) 21:408–409. doi: 10.1055/s-0031-1280842
9. Fukuhara M, Onishi S, Yonemura Y, Sato T, Tsutsumi S, Bandoh T, Utsunomiya T, Esumi G. A totally extraperitoneal approach for endoscopic resection of a congenital prepubic sinus through the pubic symphysis. Surg Case Rep (2021) 7:165. doi: 10.1186/s40792-021-01245-0
10. Duman L, Savaş Ç, Özbiçer C, Bircan S. Successful Surgical Management of Congenital Prepubic Sinus. Balkan Med J (2017) 34:88–89. doi: 10.4274/balkanmedj.2016.0538
11. Celebi S, Kuzdan Ö, Sander S, Gündüz N, Özaydın S, Yavuz S. Congenital Prepubic Sinus with Remnant Tissue Mimics Corpus Spongiosum: A Rare Case. European J Pediatr Surg Rep (2015) 3:43–45. doi: 10.1055/s-0034-1396787
12. Güler Y, Erbin A, Üçpınar B, Vural Ö, Tatar Z. Congenital prepubic sinus presenting with purulent discharge after circumcision: a case report. J Med Case Rep (2019) 13:46. doi: 10.1186/s13256-019-2019-6
13. Mostafa MS, Darwish AA. Suprapubic versus prepubic sinus: a literature review. BMJ Case Rep (2019) 12: doi: 10.1136/bcr-2019-231138
14. Ramareddy RS, Alladi A, Siddappa OS. Urethral duplication: Experience of four cases. J Indian Assoc Pediatr Surg (2012) 17:111–115. doi: 10.4103/0971-9261.98127

15. Aihole JS, Narendra Babu M, Javaregowda D, Jadhav V. Congenital prepubic sinus – A variant of urethral duplication. African Journal of Urology (2016) 22:196–198. doi: 10.1016/j.afju.2015.12.001
16. Nazir Z, Khan MAM, Qamar J. Congenital prepubic sinus-An aborted dorsal urethral duplication or a cloacal remnant? J Pediatr Surg (2019) 54:1467–1470. doi: 10.1016/j.jpedsurg.2018.07.014
17. Onofre LS, Gomes AL, Leão JQ, Leão FG, Cruz TM, Carnevale J. Urethral duplication--a wide spectrum of anomalies. J Pediatr Urol (2013) 9:1064–1071. doi: 10.1016/j.jpurol.2013.03.006
18. Abouzeid AA, Safoury HS, Mohammad SA, el-Naggar O, Zaki AM, Hassan TA, Hay SA. The double urethra: Revisiting the surgical classification. Ther Adv Urol (2015) 7:76–84. doi: 10.1177/1756287214561760
19. Wani SA, Munianjana NB, Jadhav V, Ramesh S, Gowrishankar BC, Deepak J. Urethral Duplication in Children: Experience of Twenty Cases. J Indian Assoc Pediatr Surg (2019) 24:275–280. doi: 10.4103/jiaps.JIAPS_164_18
20. Lima M, Destro F, Maffi M, D PP, Ruggeri G. Practical and functional classification of the double urethra: A variable, complex and fascinating malformation observed in 20 patients. J Pediatr Urol (2017) 13:42.e1-42.e7. doi: 10.1016/j.jpurol.2016.10.008
21. Arena S, Arena C, Scuderi MG, Sanges G, Arena F, V DB. Urethral duplication in males: our experience in ten cases. Pediatr Surg Int (2007) 23:789–794. doi: 10.1007/s00383-007-1967-x
22. Lopes RI, Giron AM, Mello MF, Neto CMB, Santos JD, Moscardi PRM, Srougi V, Denes FT, Srougi M. Urethral duplication type influences on the complications rate and number of surgical procedures. Int Braz J Urol (2017) 43:1144–1151. doi: 10.1590/S1677-5538.IBJU.2016.0269
23. Guglielmetti LC, Delcont M, Walker J, Wilcox D, Vuille-Dit-Bille RN. Urethral duplication-Epidemiology, diagnosis, and treatment in a case series of 19 patients. J Pediatr Urol (2020) 16:385.e1-385.e9. doi: 10.1016/j.jpurol.2020.02.010
24. Kang SK, Kim J, Lee YS, Han SW, Kim SW. Urethral duplication in male children: A study of 12 cases. J Pediatr Surg (2020) 55:2216–2220. doi: 10.1016/j.jpedsurg.2019.12.012
25. Wei N, Elkun Y, Ambinder D, Gitlin J, Dyer L. Congenital Anterior Urethral Diverticulum in Identical Twins. Urology (2023) 171:205–207. doi: 10.1016/j.urology.2022.09.024
26. Abdur-Rahman L, AbdulRasheed A, Nasir J, Agboola, Adeniran J. Penile shaft sinus: A sequalae of circumcision in urethral duplication. Indian Journal of Urology (2009) 25:134–136. doi: 10.4103/0970-1591.45554
27. Aksu B, Inan M, Pul M. Webbed penis associated with urethral duplication: A Case Report. Balkan Med J (2011) 28:91–93. doi: 10.5174/tutfd.2009.01497.2
28. Goyal B, Gupta S, Goyal P. Hypospadiac Duplication of Anterior Urethra—a Rare Congenital Anomaly. Indian Journal of Surgery (2017) 79:62–63. doi: 10.1007/s12262-016-1472-3
29. Ahmed N, Gul MS, Ahmed I, Rehman S, Fraz O, Anwar Z. Urethral duplication a rare cause of even rarer dorsal chordee. Pakistan Armed Forces Medical Journal (2021) 71:1124–1125. doi: 10.51253/pafmj.v71i3.5695
30. Sencan A, Yıldız M, Ergin M, Hoşgör M. A new variation of urethral duplication. Urology (2013) 82:451–453. doi: 10.1016/j.urology.2013.02.010
31. Sfoungaris D, Valioulis I, Mitroudi M, Patoulias I, Panteli C. A Boy with a Congenital Sagittal Fissure of the Glans Penis Representing an Abortive Isolated Urethral Duplication. Journal of clinical and diagnostic research (2017) 11:PJ1–PJ2. doi: 10.7860/JCDR/2017/30269.10665
32. Alanee S, Gupta P, Gleich P, Shukla AR. Complete urethral duplication: description of surgical approach mimicking distal epispadias repair. J Pediatr Urol (2012) 8:343–347. doi: 10.1016/j.jpurol.2011.06.011

33. Caione P, Angotti R, Molinaro F, Pellegrino C, Scuglia M, S GN, Messina M. Urethral duplication in male epispadias: a very uncommon association. Minerva Urol Nefrol (2020) 72:229–235. doi: 10.23736/S0393-2249.19.03271-5
34. Coleman RA, Winkle DC, Borzi PA. Urethral duplication: cases of ventral and dorsal complete duplication and review of the literature. J Pediatr Urol (2010) 6:188–191. doi: 10.1016/j.jpurol.2009.07.006
35. Mensah JE, Ampadu KN, Kyei MY, Edusie B. Bladder exstrophy associated with complete urethral duplication: Bladder can be augmented with dorsal urethral mucosa. African Journal of Urology (2013) 19:99–101. doi: 10.1016/j.afju.2013.01.003
36. Venkatramani V, George AJP, Chandrasingh J, Panda A, Devasia A. Urethral duplication with unusual cause of bladder outlet obstruction. Indian Journal of Urology (2016) 32:156–158. doi: 10.4103/0970-1591.174780
37. Roshanzamir F, Mirshemirani A, Ghoroubi J, Mahdavi A, Mohajerzadeh L, Sarafi M. Complete urethral duplication in children: A case report. Iran J Pediatr (2016) 26: doi: 10.5812/ijp.3620
38. Salimi A, Rashidinia S, Eftekhari SS, Shahmoradi S. Novel presentation of complete coronal urethral duplication: A case report. Int J Pediatr (2017) 5:4707–4712. doi: 10.22038/ijp.2017.22681.1895
39. Saxena R, Pathak M, Sinha A. Hypospadiac urethral duplication-A rare variant and novel use of Snodgrass urethroplasty. J Indian Assoc Pediatr Surg (2019) 24:138–140. doi: 10.4103/jiaps.JIAPS_62_18
40. Suoub M, Saleem MM, Sawaqed F. Complete urethral duplication: Case report and literature review. Res Rep Urol (2020) 12:15–20. doi: 10.2147/RRU.S239106
41. Uçar M, A KA, Kılıç N, Balkan E. The Association of Congenital Urethral Duplication and Double Megalourethra. Balkan Med J (2017) 34:572–575. doi: 10.4274/balkanmedj.2017.0471
42. F MTF, Birraux J, Wonkam A, Sando Z, Joko YW, Mure PY, Coultre CL, Andze GO, Sosso MA. Urethral duplication in a 12-year-old child. Afr J Paediatr Surg (2011) 8:313–316. doi: 10.4103/0189-6725.91667
43. Abraham MK, Garge S, Sudarshan B, Vishwanath N, Puzhankara R, Paliwal A, Prabhakaran A, Naaz A, Narasimhan K, Prakash D. An unusual variant of urethral duplication: an addition to the Effman classification. Int Urol Nephrol (2013) 45:601–606. doi: 10.1007/s11255-013-0424-0
44. Janssen K, Smith E, Kirsch A. Urethral Multiplicity: When Two Are Better Than One. Urology (2020) 146:216–218. doi: 10.1016/j.urology.2020.06.074
45. Mane SB, Obaidah A, Dhende NP, Arlikar J, Acharya H, Thakur A, Reddy S. Urethral duplication in children: our experience of eight cases. J Pediatr Urol (2009) 5:363–367. doi: 10.1016/j.jpurol.2009.01.006
46. Lena F, Pellegrino C, Zaccara AM, Capitanucci ML, Esposito G, Iacobelli BD, Longo D, Caldaro T, Bruno D, Bevilacqua F, et al. Anorectal malformation, urethral duplication, occult spinal dysraphism (ARM-UD-OSD): a challenging uncommon association. Pediatr Surg Int (2022) 38:1487–1494. doi: 10.1007/s00383-022-05186-z
47. Baid M, Dutta A. Urethral Duplication in a 15-Year-Old: Case Report With Review of the Literature. Rev Urol (2014) 16:149–151.
48. Davidson JR, Wright NJ, Garriboli M. Urethral Duplication with Two Hypospadic Meati—An Unusual Variant. European J Pediatr Surg Rep (2016) 4:37–40. doi: 10.1055/s-0036-1588015
49. Shah DH, Ganpule AP, Mishra SK, Sabnis RB, Desai MR. Congenital duplication of urethra with urethral diverticulum: a rare case report. BJU Int (2014) 114:20–21.
50. Alhasan S. Urethral duplication. Ann Afr Med (2012) 11:190. doi: 10.4103/1596-3519.96883

51. Gupta A, Pant N, Debnath PR, Yadav PS, Agarwala SK, Saxena R, Chadha R. Type IIA2 urethral duplication: Report of an unusual case. Annals of Pediatric Surgery (2015) 11:162–165. doi: 10.1097/01.XPS.0000459985.72968.77
52. Freitas Filho LG, Martins CS, Carnevale J, Kanasiro F, Budib LJ. Spinal lipoma associated with urethral duplication. Urol Case Rep (2018) 19:11–12. doi: 10.1016/j.eucr.2018.04.002
53. Polukhov R, Mahammadov V, Baghirli M. Epispadias associated with urethral duplication: Our practice. Int J Surg Case Rep (2020) 71:199–201. doi: 10.1016/j.ijscr.2020.05.002
54. Mazaheri T, Rad M V, Fareghi M, Kajbafzadeh AM. Multiple hypoplastic duplicated urethral hydrodistension and simple end-to-end anastomosis to penoscrotal hypospadias: a novel technique. Int Urol Nephrol (2014) 46:1729–1731. doi: 10.1007/s11255-014-0740-z
55. Yanai T, Kawakami H, Nango Y, Watayo H, Masuko T, Hirai M, Muraji T. Minimally invasive repair of hypospadiac urethral duplication. Pediatr Surg Int (2011) 27:115–118. doi: 10.1007/s00383-010-2720-4
56. Danish N, Ansari MS, Lal H, Yadav P. Urethral Duplication With Heterotopic Colonic Epithelium Presenting as Penoscrotal Mass: A Rare Association. Urology (2020) 142:200–203. doi: 10.1016/j.urology.2020.04.050
57. Ragavan M, Uppalu H, Prem S, Sarvavinothini J. Patent urachus with double urethra and testicular teratoma: A rare association. J Indian Assoc Pediatr Surg (2011) 16:108–110. doi: 10.4103/0971-9261.83495
58. Sindjic S, Perovic S V, Djinovic RP. Complex case of urethral duplication with megalourethra. Urology (2009) 74:903–905. doi: 10.1016/j.urology.2008.12.040
59. Singh S, Rawat J. Y-type urethral duplication in children: Management strategy at our center. J Indian Assoc Pediatr Surg (2013) 18:100–104. doi: 10.4103/0971-9261.116042
60. Taghavi K, Trachta J, Mushtaq I. Urethral duplication: A case for careful examination. Arch Dis Child (2019) 104:685. doi: 10.1136/archdischild-2018-314830
61. Saran R, Mirdha K, Saran S, Takhar R. Urethral duplication with rectourethral fistula: Review of two cases. Urol Ann (2020) 12:92–95. doi: 10.4103/UA.UA_25_19
62. Danzig MR, Kryger J V, Groth TW, Roth EB, Ellison JS. Technical details and long-term outcomes of P.A.D.U.A. for congenital urethral narrowing; a case series and review of the literature. J Pediatr Urol (2022) doi: 10.1016/j.jpurol.2022.10.030
63. Jr MA, Silva MIS, Pompermaier JA, Ottoni SL, R de C, M L da C. The anterior sagittal transrectal approach (ASTRA) for cases associated with rectal implantation of the urethra: A retrospective review of six cases. J Pediatr Urol (2017) 13:613.e1-613.e4. doi: 10.1016/j.jpurol.2017.04.011
64. Raffoul L, Rod J, Ravasse P, Blanc T, Lortat-Jacob S. Q-island flap urethroplasty: 1-stage procedure for reconstruction of Y-type urethral duplications in children. J Urol (2015) 193:2068–2072. doi: 10.1016/j.juro.2015.01.082
65. Kurian JJ, Thomas JK, Kuppusamy S, Kisku S, Arunachalam P, Sen S. Working classification and the quest for an effective, reconstructive management strategy in Y duplication of male urethra. J Pediatr Urol (2021) 17:414.e1-414.e8. doi: 10.1016/j.jpurol.2021.02.008
66. Lorenz C, Zahn K, Schäfer FM, Möller K, Stehr M, Stein R. Congenital Y-urethra - A diagnostic and therapeutic challenge. J Pediatr Urol (2021) 17:30–38. doi: 10.1016/j.jpurol.2020.11.032
67. AbouZeid AA, Mohammad SA, Radwan NA, Safoury HS, El-Naggar O, Hay SA. Y-Type Urethral Duplication: A True Variant of the Anomaly or a Misnomer? Eur J Pediatr Surg (2016) 26:245–251. doi: 10.1055/s-0035-1551569

68. Lima M, Destro F, N DS, Gargano T, Ruggeri G. Fate of males with urethral “Y-duplication”: 40-year long follow-up in 8 patients. J Pediatr Surg (2017) 52:1335–1339. doi: 10.1016/j.jpedsurg.2016.11.034
69. Zhang Y, Qu Y, Jiao L, Zhang W, Sun N, Tian J, Li M, Song H. Urodynamic performance in boys with Y-type urethral duplication. J Pediatr Surg (2018) 53:1326–1329. doi: 10.1016/j.jpedsurg.2017.06.001
70. Pati AB, Sahoo SK, Tripathy BB. Progressive Dilatation as a Successful Treatment for Y Duplication of Urethra: A case report. Sultan Qaboos Univ Med J (2023) 23:119–121. doi: 10.18295/squmj.9.2021.138
71. Meier DE, Latiff A. Y-type congenital urethral duplication with normal dorsal urethra and small ventral fistula to perineal skin - 28th reported case. J Pediatr Surg Case Rep (2016) 8:37–39. doi: 10.1016/j.epsc.2016.03.008
72. Nonnenmacher G, Alazki A, Wang S, Loff S. Experiences in management of a Y-type urethral duplication. J Pediatr Surg Case Rep (2021) 72: doi: 10.1016/j.epsc.2021.101981
73. Deeb MA, Gawrieh B, Hemi F, Alelayan AF, Omran A. An unusual Y-Type urethral duplication in a boy. J Pediatr Surg Case Rep (2022) 78: doi: 10.1016/j.epsc.2021.102169
74. Bello JO. Congenital posterior urethroperineal fistula: a review and report of the 25th case in literature. Urology (2014) 84:1492–1495. doi: 10.1016/j.urology.2014.09.002
75. Dayanc M, Irkilata HC, Kibar Y, Bozkurt Y, Basal S, Xhafa A. Y-type urethral duplication presented with perineal fistula in a boy. Ger Med Sci (2010) 8:Doc33-. doi: 10.3205/000122
76. Jr MA, L da CM, Parizi JLG, Martins GMC, Liguori R, Ottoni SL, Leslie B, Garrone G. An unusual presentation of urethral duplication presenting with chronic bladder retention, left scrotal transposition and left renal agenesis. Int Braz J Urol (2018) 44:409–410. doi: 10.1590/S1677-5538.IBJU.2016.0119
77. Jr MA, Ottoni SL, M L da C, Pompermaier JA, Silva MIS, Liguori R, Garrone G. Y-type urethral duplication with rectal implantation of the urethra: Which is the best approach? J Pediatr Urol (2018) 14:79–80. doi: 10.1016/j.jpurol.2017.10.009
78. Chowdhary S, Sharma SP. A new anatomic variant of urethral duplication. J Pediatr Surg Case Rep (2018) 33:74–76. doi: 10.1016/j.epsc.2018.04.008
79. Hanci B, Dincer E, Karaca Y, Bulut M, Sevinc A, Ozcan T, Cetinel AC, Telli O. A Rare Variant of Urethral Duplication: Type-3 Bladder and Complete Urethral Duplication. JOURNAL OF UROLOGICAL SURGERY (2020) 7:70–71. doi: 10.4274/jus.galenos.2019.2837
80. Coker AM, Allshouse MJ, Koyle MA. Complete duplication of bladder and urethra in a sagittal plane in a male infant: case report and literature review. J Pediatr Urol (2008) 4:255–259. doi: 10.1016/j.jpurol.2008.02.001
81. Pirinççi N, Geçit I, Güneş M, Tanık S, Ceylan K. Complete duplication of the bladder and urethra in the coronal plane: case report with review of the literature. Urol Int (2013) 90:118–120. doi: 10.1159/000339922
82. Parente A, Ortiz R, Burgos L, Angulo JM. Urethral Triplication Without Bladder Duplication: Endourologic Diagnosis andManagement. J Endourol Case Rep (2018) 4:45–47. doi: 10.1089/cren.2018.0019
83. Lin HW, Shen XW, Geng HQ, Xu MS. Treatment and Medical Follow-up of a Boy With Urethral Triplication. Urology (2012) 80:214–215. doi: 10.1016/j.urology.2012.03.031
84. Kajbafzadeh AM, Taleb S, Montaser-Kouhsari L, Tanhaeivash R, Monajemzadeh M, Mehdizadeh M. Urethral Triplication and Urethrovasal Reflux in 5-Day-old Male Infant. Urology (2011) 78:186–188. doi: 10.1016/j.urology.2010.09.019

85. Kurian JJ, Homi SJ, Karl IS. A rare case of urethral triplication in association with tethered cord and vertebral anomalies. Annals of Pediatric Surgery (2016) 12:119–121. doi: 10.1097/01.XPS.0000469366.65557.5e
86. Zhang Z, Xu Y, He L, Li Y, Zhang J, Fang X, Xu M, Geng H, Xu G. Urethral Triplication With Diverticulum Malformation: A Case Report and Literature Review. Urology (2020) 144:198–201. doi: 10.1016/j.urology.2020.05.053
87. Hirselj DA, Lowe GK, Jayanthi VR. Urethral triplication with meatuses terminating on the penis: a rare case presenting with bifid urinary stream. Pediatr Radiol (2009) 39:1227–1229. doi: 10.1007/s00247-009-1345-z
88. Bowen DK, Glaser AP, Bush JW, Cheng EY, Gong EM. Combined robotic and open approach to excision of accessory bladder and urethral triplication. J Pediatr Urol (2015) 11:98–99. doi: 10.1016/j.jpurol.2014.11.009
89. Tourchi A, Kajbafzadeh AM, Khakpour M, P MN, Mousavian AA, Kalantary M. Concomitant urethral triplication, bladder, and colon duplication. Int Urol Nephrol (2012) 44:41–44. doi: 10.1007/s11255-011-9981-2
90. Maitama HY, Mbibu HN, Tella UM. Urethral duplication: case report and literature review. Ann Afr Med (2012) 11:186–189. doi: 10.4103/1596-3519.96883
